# Exploring a Nonmodel Teleost Genome Through RAD Sequencing—Linkage Mapping in Common Pandora, *Pagellus erythrinus* and Comparative Genomic Analysis

**DOI:** 10.1534/g3.115.023432

**Published:** 2015-12-28

**Authors:** Tereza Manousaki, Alexandros Tsakogiannis, John B. Taggart, Christos Palaiokostas, Dimitris Tsaparis, Jacques Lagnel, Dimitrios Chatziplis, Antonios Magoulas, Nikos Papandroulakis, Constantinos C. Mylonas, Costas S. Tsigenopoulos

**Affiliations:** * Institute of Marine Biology, Biotechnology and Aquaculture, Hellenic Centre for Marine Research, Heraklion, 71500, Greece; † Department of Biology, University of Crete, Heraklion, 70013, Greece; ‡ Institute of Aquaculture, School of Natural Sciences, University of Stirling, Scotland, FK9 4LA, United Kingdom; § Laboratory of Agrobiotechnology and Inspection of Agricultural Products, Department of Agricultural Technology, School of Agricultural Technology Food Technology and Nutrition, Alexander Technological Educational Institute of Thessaloniki, Sindos, 57400, Greece

**Keywords:** Sparidae, RAD sequencing, ddRAD, synteny, aquaculture

## Abstract

Common pandora (*Pagellus erythrinus*) is a benthopelagic marine fish belonging to the teleost family Sparidae, and a newly recruited species in Mediterranean aquaculture. The paucity of genetic information relating to sparids, despite their growing economic value for aquaculture, provides the impetus for exploring the genomics of this fish group. Genomic tool development, such as genetic linkage maps provision, lays the groundwork for linking genotype to phenotype, allowing fine-mapping of loci responsible for beneficial traits. In this study, we applied ddRAD methodology to identify polymorphic markers in a full-sib family of common pandora. Employing the Illumina MiSeq platform, we sampled and sequenced a size-selected genomic fraction of 99 individuals, which led to the identification of 920 polymorphic loci. Downstream mapping analysis resulted in the construction of 24 robust linkage groups, corresponding to the karyotype of the species. The common pandora linkage map showed varying degrees of conserved synteny with four other teleost genomes, namely the European seabass (*Dicentrarchus labrax*), Nile tilapia (*Oreochromis niloticus*), stickleback (*Gasterosteus aculeatus*), and medaka (*Oryzias latipes*), suggesting a conserved genomic evolution in Sparidae. Our work exploits the possibilities of genotyping by sequencing to gain novel insights into genome structure and evolution. Such information will boost the study of cultured species and will set the foundation for a deeper understanding of the complex evolutionary history of teleosts.

The Sparidae is a species-rich family of marine teleosts belonging to the Percomorphs group. Sparids inhabit tropical and temperate coastal waters. They are of considerable economic importance, particularly around the Mediterranean area, with many species being targeted by capture fisheries, and a lesser number also being cultured commercially (Basurco *et al.* 2011). Currently, the dominating species in Mediterranean aquaculture is gilthead seabream (*Sparus aurata*), the most intensively studied sparid. However, driven by the need for diversification within the aquaculture industry, other sparid species have been gaining in commercial and scientific interest. This group exhibits extensive variability in reproductive modes, such as alternative types of hermaphroditism and gonochorism (Mylonas *et al.* 2011), with contrasting mechanisms being found even among closely related species (Erisman *et al.* 2013). As such, they are gaining their own “niche” in the new model fish army (Braasch *et al.* 2014) for studying the evolution of hermaphroditism.

One of the baselines for efficient genetic selection programs in any species is the availability of genetic linkage maps. Linkage maps allow for mapping phenotypic traits of interest and provide a backbone for further genetic studies up to whole genome sequencing. Until recently, building a map generally required the genotyping of hundreds of microsatellite markers across a genome. The newly developed genotyping by sequencing technologies, which allow the *de novo* discovery and simultaneous scoring of hundreds to thousands of single nucleotide polymorphism (SNP) markers from a single sequencing run for dozens of individuals, provide a new means to rapidly characterize the genomes of nonmodel species. Various adaptations of these genome-reduction screening techniques provide alternative approaches for different applications. RAD-Seq (Baird *et al.* 2008) is one of the earliest described methodologies that allows the routine identification of many thousands of SNPs, but requires considerable sequencing effort per individual. Variations of this method [*e.g.*, Genotyping-by-Sequencing (Elshire *et al.* 2011); ddRAD (Peterson *et al.* 2012); 2bRAD (Wang *et al.* 2012); ezRAD (Toonen *et al.* 2013); SLAF-Seq (Sun *et al.* 2013); and GT-Seq (Campbell *et al.* 2015)] can be employed to limit the extent of marker discovery to a lesser, but adequate, degree, thereby allowing sequencing of a greater number of individuals for fewer markers for the same sequencing effort. Linkage maps using genotyping by sequencing approaches have already been produced for numerous fishes, such as the spotted gar (*Lepisosteus oculatus*) (Amores *et al.* 2011), Midas cichlid (*Amphilophus spp*.) (Recknagel *et al.* 2013), gudgeon (genus *Gnathopogon*) (Kakioka *et al.* 2013), blind cavefish (*Astyanax mexicanus*) (O’Quin *et al.* 2013), Nile tilapia (*Oreochromis niloticus*) (Palaiokostas *et al.* 2013a), Atlantic halibut (*Hippoglossus hippoglossus*) (Palaiokostas *et al.* 2013b), orange-spotted grouper (*Epinephelus coioides*) (You *et al.* 2013), Japanese eel (*Anguilla japonica*) (Kai *et al.* 2014), and platyfish (*Xiphophorus maculatus*) (Amores *et al.* 2014), among others. The construction of relatively dense linkage maps without prior knowledge of genetic marker panels sets a new standard for studying nonmodel species.

Most of the Mediterranean sparids have 24 haploid chromosomes (Cataudella *et al.* 1980), in line with the majority of teleosts (Naruse *et al.* 2004; Galetti *et al.* 2000). The family member that has been most rigorously studied in terms of its genetics is gilthead seabream. Previous efforts have produced two radiation hybrid maps (Senger *et al.* 2006; Sarropoulou *et al.* 2007), one BACmap (Kuhl *et al.* 2011), and two genetic maps based on microsatellites (Franch *et al.* 2006; Tsigenopoulos *et al.* 2014). Linkage mapping efforts have allowed also for quantitative trait locus (QTL) mapping (Boulton *et al.* 2011; Loukovitis *et al.* 2011, 2012, 2013; Massault *et al.* 2011) and are expected to facilitate the ongoing genome sequencing project of gilthead seabream.

Until recently, with the exception of gilthead seabream, relatively limited effort has been invested in exploring the genomic background of sparids. To our knowledge, recent work on the sex-specific transcriptomic profiling for the rudimentary hermaphrodite sharpsnout seabream (*Diplodus puntazzo*) (Manousaki *et al.* 2014) is the only report on another sparid. Here, we focus on the protogynous common pandora (*Pagellus erythrinus*), a benthopelagic sparid found across the Mediterranean and North East Atlantic. Although only recently farmed commercially, production is increasing year by year, and its potential as a significant aquaculture species is recognized (Basurco *et al.* 2011). Comparatively little is known about the genetics of common pandora, with only a few studies reporting on the use of genetic markers to study population structure within the species (Apostolidis *et al.* 2009; Fassatoui *et al.* 2009, 2012). In the present study, we employed double digest restriction associated DNA (ddRAD) sequencing to construct the first genetic linkage map for common pandora. We identified nearly 1000 polymorphic ddRAD loci, and built a genetic linkage map comprising 24 linkage groups (LGs). Furthermore, comparative analyses uncovered homologies between common pandora and four other ‘model’ fish species, namely the European seabass (*Dicentrarchus labrax*), Nile tilapia (*Oreochromis niloticus*), stickleback (*Gasterosteus aculeatus*), and medaka (*Oryzias latipes*). Finally, through a phylogenetic analysis we provided insights into the phylogenetic relationships among these species.

## Materials and Methods

### Ethics statement

All experiments were performed in accordance with the “Guidelines for the treatment of animals in behavioral research and teaching” (Animal Behavior Society 2001).

### Selection of linkage panel

A wild-caught common pandora broodstock was maintained in culture conditions at the AQUALABS facility of the Hellenic Centre for Marine Research (HCMR), Crete, Greece. The fish were exposed to simulated ambient photo-thermal conditions and were allowed to spawn spontaneously. A male and a female fish were kept separately, and spawned at the end of June 2013. The floating eggs were collected and reared in a mesocosm using commercial larval rearing methods for sparid fishes. Fin clips were sampled from the two parents, and 2 months later from 97 of their offspring (average weight approximately 1.5 g). Samples were kept at –20° until DNA extraction. DNA was extracted by a modified salt-based extraction protocol using SSTNE extraction buffer (Blanquer 1990), and treated with RNase to remove residual RNA. Genomic DNA was eluted in 5 mmol Tris, pH 8.5 and stored at 4°. Each sample was quantified by spectrophotometry (Nanodrop 1000, Thermo Fisher Scientific) and quality assessed by 0.7% agarose gel electrophoresis. Parents, together with 97 full-sibs, were used for the ddRAD library construction (99 fish in total).

### ddRAD library preparation and sequencing

The ddRAD library preparation protocol was based on the methodology originally reported by Peterson *et al.* (2012). The modified protocol used here is essentially that described in Palaiokostas *et al.* (2015), with additional refinements being flagged below. Briefly, each of 103 separate DNA samples (both parents in triplicates and 97 offspring; 20 ng DNA per sample) was simultaneously digested by two high fidelity restriction enzymes (RE): *SbfI* (CCTGCA|GG recognition site), and *SphI* (GCATG|C recognition site), both sourced from New England Biolabs, (NEB) UK. Digestions were incubated at 37° for 50 min, using 10 U of each enzyme per microgram DNA in 1× CutSmart Buffer (NEB), in a 6 μl total reaction volume. Deviating from the methodology described in Palaiokostas *et al.* (2015), the reactions were not heat-inactivated as this was deemed to be unnecessary, and possibly detrimental, given the high temperature (80°) recommended by the RE supplier. Barcoded adapters were designed such that adapter–genomic DNA ligations did not reconstitute RE sites, while residual RE activity limited concatemerization of genomic fragments. After cooling the reactions to room temperature, 3 μl of a premade adapter mix was added to the digested DNA, and incubated at room temperature for 10 min. This adapter mix comprised individual-specific combinations of P1 (*Sbf*I-compatible) and P2 (*Sph*I-compatible) adapters at 6 nM and 72 nM concentrations respectively, in 1× reaction buffer 2 (NEB). The ratio of P1 to P2 adapter (1:12) was different than that described previously (1:4; Palaiokostas *et al.* 2015), as this was expected to more accurately reflect the relative abundance of *Sbf*I and *Sph*I cut sites present. P1 and P2 adapter included an inline five- or seven-base barcode for sample identification. Ligation was performed over 3 hr at 22° by addition of a further 3 µl of a ligation mix comprising 4 mM rATP (Promega, UK), and 2000 cohesive-end units of T4 ligase (NEB) in 1× CutSmart buffer (NEB). The ligated samples were then heat-denatured at 65° for 20 min, cooled, and combined into a single pool. The pooled sample was column-purified (MinElute PCR Purification Kit, Qiagen, UK), and eluted in 100 μl EB buffer (Qiagen, UK). Size selection of fragments, ranging from approximately 300 bp to 600 bp, was performed by agarose gel separation. Following gel purification (MinElute Gel Extraction Kit, Qiagen, UK), the eluted size-selected template DNA (60 μl in EB buffer) was PCR amplified (14 cycles PCR; 36 separate 12.5-μl reactions, each with 1 µl template DNA) using a high fidelity *Taq* polymerase (Q5 Hot Start High-Fidelity DNA Polymerase, NEB). The PCR reactions were combined (450 μl total), and column-purified (MinElute PCR Purification Kit). The 55-μl eluate, in EB buffer, was then subjected to a further size-selection clean-up using an equal volume of AMPure magnetic beads (Perkin-Elmer, UK), to maximize removal of small fragments (less than *ca*. 200 bp). The final library was eluted in 22 μl EB buffer. Finally, the ddRAD library was sequenced at the Institute of Marine Biology, Biotechnology and Aquaculture (IMBBC) of HCMR in Crete using two runs of an Illumina MiSeq (v2 chemistry, 300 cycle kit, 162 bp paired end reads).

### Building RAD loci

Raw reads were analyzed in Stacks 1.19 (Catchen *et al.* 2011). Quality control, filtering for ambiguous barcodes and restriction sites, and demultiplexing took place using the script *process_radtags* (options -c -q -r). Due to the use of barcodes with different length, and limitations in this Stacks version to accommodate this design in a single step, *process_radtags* took place in four independent steps. First, the samples with seven-base-long barcodes in both ends were demultiplexed, second those with five at one end and seven bases at the other, third those with seven at one end and five at the other, and finally those with five-base barcodes at both ends. After each demultiplexing step, the unassigned reads (including the reads with the barcode combinations other than that in the ongoing step) were reconstructed in pairs using the script *fastqCombinePairedEnd.pl* written by Eric Normandeau (https://github.com/enormandeau/Scripts/blob/master/fastqCombinePairedEnd.py), and used for the subsequent round of demultiplexing.

The files containing the paired forward and reverse reads of each sample were then concatenated and reads were trimmed to a length of 100 bases. Reads less than 100 bases long were discarded. Trimming and filtering took place with FASTX_toolkit (http://hannonlab.cshl.edu/fastx_toolkit/). Stacks were built for each individual with the wrapper script *denovo_map.pl* included in Stacks using the default parameters (a minimum of three reads to form a stack, and a minimum of two mismatches allowed between loci when processing a single individual). Secondary reads were not used for genotype calling to reduce possible genotypic errors (option -H).

### Linkage map construction

For linkage mapping, we exported the haplotypes of each individual for each RAD locus using the Stacks script *genotypes* in the OneMap format (-r 80 -t CP -o onemap -c -s). Only parental loci mapped in more than 80 out of 97 progeny were kept. Finally, loci showing significant segregation distortion (*chi*-square *p* value < 0.01) were excluded. The resultant genotypic data were input in OneMap (Margarido *et al.* 2007)—a software for genetic mapping in outbred populations. OneMap applies the methodology proposed by Wu *et al.* (2002), and leads to the construction of a linkage map combining information from markers showing different segregation patterns in both parents. Recombination fraction between all pairs of markers was estimated with two-point tests (function *rf.2pts*). Then, markers were grouped to linkage group with the function *group*. To select the appropriate LOD score, we used a range of LOD scores incrementing by one and starting from the value three up to ten (Supporting Information, Figure S1) with a maximum recombination fraction of 0.3. The final LOD score for marker grouping was selected based on whether the number of LGs matched the number of chromosomes of common pandora. Then, markers within each linkage group were ordered using the *order.seq* function within OneMap (n.init = 5, THRES = 4), which conducts an exhaustive search for the five most informative markers, and then adds one marker at a time with a minimum LOD score 4. With the *order.seq* function, markers that were not uniquely mapped were mapped on the most likely position (the one with the largest value of log-likelihood). Following marker ordering, alternative orders were checked with the function *ripple.seq*. Map distances were calculated using the Kosambi map function (see File S1 for the R script including the commands implemented in OneMap). LGs were numbered based on the homology with European seabass, the species with the highest similarity as shown by the comparative genomic analyses presented in the *Results* section. Finally, the map was visualized with MapChart (Voorrips 2002).

### Comparative genomics

The mapped RAD loci in common pandora were used in a comparative analysis with genomes from the following relatively closely related teleosts: medaka (*Oryzias latipes*, Ensembl 73), stickleback (*Gasterosteus aculeatus*, Ensembl 73), Nile tilapia (*Oreochromis niloticus*, ncbi GCA_000188235.2) and European seabass (*Dicentrarchus labrax*, dicLab v1.0c http://seabass.mpipz.de). Common pandora RAD loci sequences were extracted from the MySQL database built through Stacks pipeline, and were used in BLASTN sequence similarity searches against the genomes of the four other species (*e*-value threshold 10^−9^). Loci with more than 10 hits or more than 10 HSPs (high-scoring segment pairs) within the first hit, were excluded to eliminate repetitive sequences. The top hit per sequence was retained and considered homologous to the RAD locus. The chromosome of the reference species that corresponded to each common pandora LG was inferred based on the similarity search. If most loci of a common pandora LG were homologous to loci from a single chromosome in a reference species, we considered them homologous chromosomes. All links between common pandora LGs and the reference species chromosomes were displayed with Circos software (Krzywinski *et al.* 2009). To investigate which loci are located within protein coding sequences, we conducted a second round of similarity searches against each of the four species cDNA sequence datasets, downloaded from http://seabass.mpipz.de for European seabass with corresponding annotations, and Ensembl 73 for the other three, and ran with the same parameters as above. The numbers of shared hits found in the four species were represented by Venn diagrams constructed using the online tool available at http://bioinformatics.psb.ugent.be/webtools/Venn/.

Finally, to check whether the number of hits found in each of the four species was due to variable genome sequencing completeness, we implemented the CEGMA pipeline (Parra *et al.* 2007), and assessed the completeness based on the percentage of 248 conserved genes present even partially in each genome.

### ddRAD-based phylogenetic analysis

From the comparative genomic analysis we selected the loci showing hits in all four species after excluding those whose hits overlapped even partially in any of the four genomes. The homologous loci sequences from each species were extracted with a custom perl script. Then, all sequences were aligned for each locus independently using mafft v7.050b (–auto option) (Katoh and Standley 2013). The individual alignments were concatenated to a matrix with the perl script catfasta2phyml.pl (available at https://www.abc.se/~nylander/catfasta2phyml/). The matrix was filtered to eliminate divergent and ambiguously aligned regions with GBlocks v 0.91b (Castresana 2000) (default parameters apart from setting the maximum number of contiguous nonconserved positions to five). The resultant filtered alignment was used for phylogenetic analysis. First, the model of nucleotide substitution was chosen with jModelTest 2.1.7 (Guindon and Gascuel 2003; Darriba *et al.* 2012). Then, the best model returned through Akaike *information criterion* (AIC) and *Bayesian information criterion* (BIC) criteria was used to estimate the tree topology in PhyML 3.1 (Guindon and Gascuel 2003). Branch support was based on 100 bootstrap (BS) datasets.

### Data availability

Raw reads are deposited in N.C.B.I. sequence read archive under the BioProject ID PRJNA302241.

## Results

### ddRAD data analysis

Following initial quality filtering, demultiplexing, and length filtering of the two combined MiSeq runs, a total of 40,662,844 high quality reads were assigned to the 99 individuals (Table S1). As planned, a higher coverage was obtained for the two parents (in excess of 2 million reads each), while read numbers for progeny ranged from ∼89,000 to 1.05 million reads (mean 366,861; SD 185,182). Stacks analysis identified 10,821 and 11,657 RAD loci in sire and dam respectively (mean coverage per locus in excess of ×180), with 2647 and 2591 potential SNPs being identified. Progeny contained on average 8211 stacks (SD 4106) with an average coverage of ×36 per locus. The number of potential SNPs identified in the progeny ranged from 1267 to 2328 (mean 1949; SD 223). Finally, out of 2947 parental loci cataloged that contained one or two SNPs, 1032 were genotyped in at least 80 progeny. Loci that exhibited significant segregation distortion were discarded, resulting in 920 RAD loci containing 1181 SNPs that were used in the subsequent linkage analysis (Table S2).

### Common pandora linkage map

**Figure 1:**
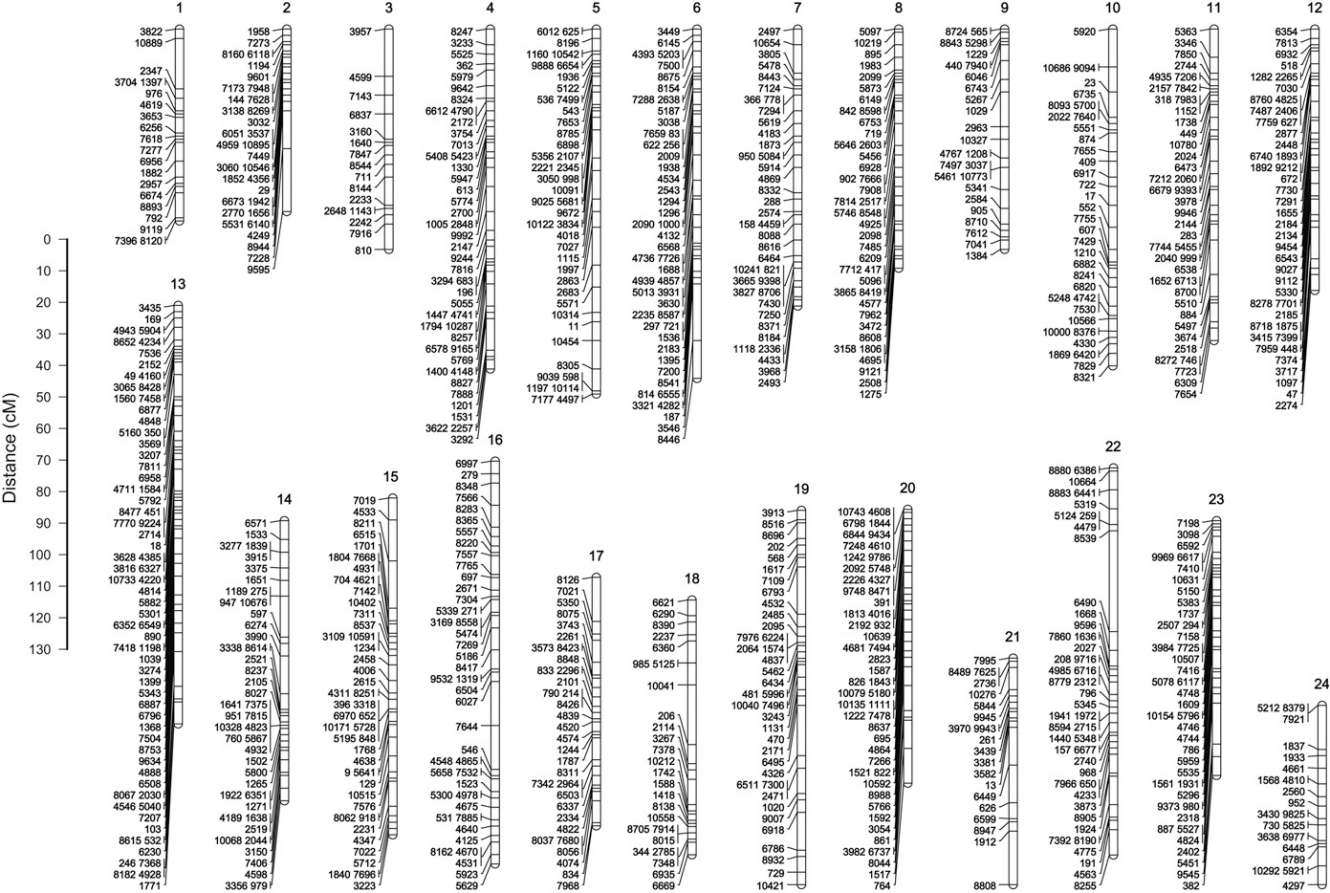
Linkage map of common pandora *Pagellus erythrinus* produced from 917 ddRAD loci. Map distances are calculated using the Kosambi function. The black lines indicate the location of the markers on the linkage groups. Numbering follows this in European seabass based on synteny analyses.

The 920 informative RAD loci comprised the following segregation patterns: aa/ab 323, ab/ab 99, ab/aa 352, and ab/ac 146. For the linkage mapping, we conducted a thorough exploration of LOD scores to recover a linkage map that approaches the species karyotype (see *Materials and Methods*). We selected the LOD value six, which resolved 24 linkage groups (Figure S1), and corresponds to the haploid chromosome number of common pandora (Cataudella *et al.* 1980). The constructed LGs, incorporating 917 of the 920 identified markers, contained from 16 to 71 RAD loci spanning 56.63 to 132.54 cM in length (Figure 1 and Table S2). The total length of the map was 2201.78 cM. After conflating markers that occurred at same position at the linkage map, 686 unique mapping positions were identified, with a mean distance between them of 3.98 cM.

### Comparative genomic analysis

**Figure 2:**
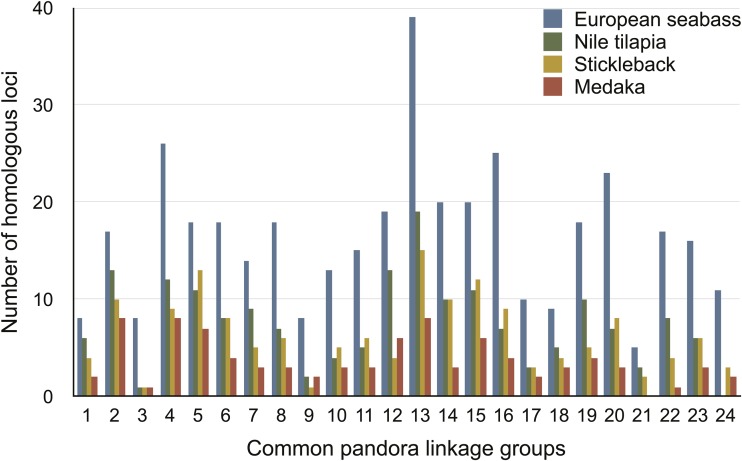
The number of homologous loci for each of common pandora linkage group with European seabass, Nile tilapia, stickleback, and medaka.

To validate the constructed linkage map and compare the genomic architecture of common pandora to that of other teleosts, we identified the most similar homologous regions of common pandora RAD loci present in the genomes of medaka, stickleback, Nile tilapia and European seabass (*e*-value threshold 10^−9^) (Figure 2 and Table S2). This search revealed numerous presumed homologous loci in each of those four species (Table 1 and Table 2). The majority of the ‘homologous’ loci identified were positioned on chromosomes and many of them fell within coding sequences (Table 2, Table S2, and Figure S3). The remaining loci were located in unlinked scaffolds or contigs, and were excluded from downstream analyses. To test whether the difference in the number of homologous loci in the four model species might be a reflection of the genomic coverage, we identified and compared the CEGMA core genes within their genomes. The results showed that Nile tilapia genome contains, at least partially, 98.39% of CEGMA core genes, stickleback genome contains 97.50%, medaka contains 97.98% and European seabass contains 97.98%, suggesting a similar coverage over all these genomes.

**Table 1:** Summary of the genetic linkage map of common pandora, and comparative analysis with the chromosomes of medaka, stickleback, Nile tilapia, and European seabass

Common Pandora Map Summary			Homologous Chromosomes			
LG	Number of Loci	Length (cM)	European Seabass	Nile Tilapia	Stickleback	Medaka
1	20	60.7	1A	5	XVII	5
2	33	57.61	2	2	IV	10
3	16	70.1	3/14	3	VII	18
4	46	108.31	4	23	VIII	4
5	44	116.39	5	1	II	3
6	49	110.57	6	7	XIX	6
7	39	87.84	7	6	IX	1
8	41	76.31	8	4	XI	8
9	26	69.82	9	22	X	11
10	35	106.52	10	18	III	17
11	42	99.15	11	13	VI	15
12	44	82.85	12	19	XV	22
13	71	132.54	13	14	I	13
14	45	89.38	14	10	VII	14
15	46	107.25	15	16	XVI	21
16	44	127.83	1B/16	11	XX	16/19/2/3
17	33	79.25	17	15	XVIII	24
18	26	80.76	18	9	XXI	20
19	38	118.93	19	7	XIV	12
20	51	86.65	20	12	XIII	9
21	20	71.71	1B	8	V	—
22	46	123.32	22	20	XII	7
23	41	81.36	X	17	IV	23
24	21	56.63	24	—	I	2

**Table 2:** Summary of common pandora linkage map comparative analysis***^a^***

Species	Total Number of Homologs	Number of Homologs in Chromosomes	Number of Homologs in Coding Regions
Medaka	96	89	60
Stickleback	167	153	83
Nile tilapia	215	180	76
European seabass	420	395	130

a Note: See Figure S3 for shared loci among species.

**Figure 3:**
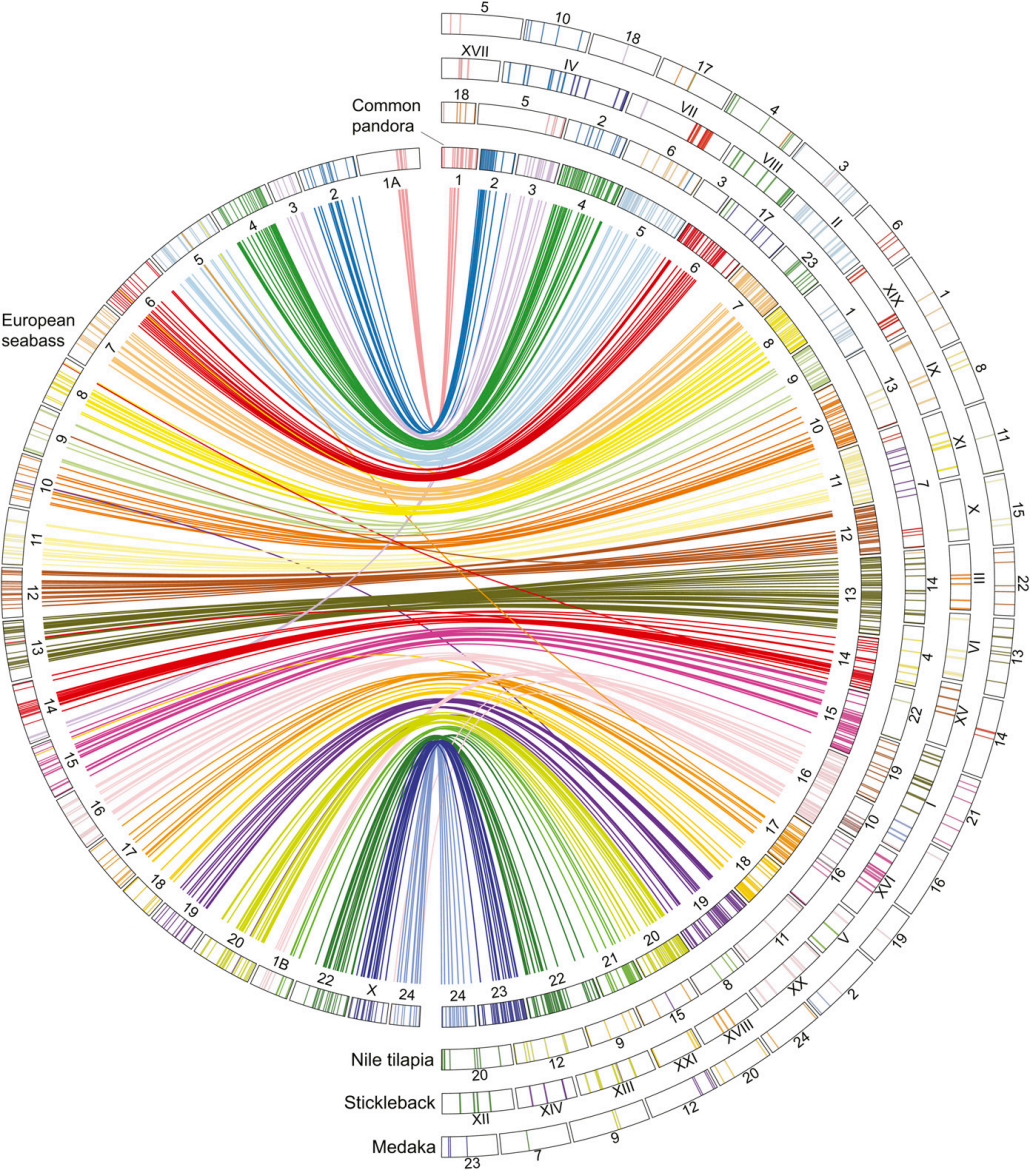
Comparative view of common pandora linkage groups with European seabass. Common pandora linkage groups are named based on their homologous European seabass chromosome. At the periphery, from inner to outside, the homologous loci of common pandora with Nile tilapia, stickleback, and medaka are shown. Bands show the loci position, and their color represents the different common pandora linkage groups.

The RAD loci identified as having significant sequence similarities within the chromosomes/linkage groups of medaka, stickleback, Nile tilapia, and European seabass were used in the comparative analysis (Table 1). Inferring the genomic location of the homologous loci in all four species revealed extensive conservation of synteny (Figure 3 and Figure S2). The highest degree of similarity was detected with European seabass, with which 43% of common pandora RAD loci were matched, fewer in Nile tilapia (20%) and stickleback (17%), and least in medaka (10%).

At the linkage group level, the comparison with medaka (*n* = 24) revealed one-to-one homology with the great majority of common pandora LGs (peLG) (Table 1). Where homology with medaka chromosomes (olChr) was found, up to eight ‘homologous’ loci per peLG were identified. Although all medaka chromosomes had hits with common pandora loci, peLG21 showed no homology with any medaka chromosome. Further, common pandora peLG16 appeared to have partial homology with four medaka chromosomes, making assignment of homology unclear. The remainder of peLGs resolved to a potentially homologous single medaka chromosome (Table 1), though this can only be stated with limited confidence due to the relatively low number of homologous loci found between the two species.

The comparison with stickleback (*n* = 21) also revealed one-to-one homology for the great majority of peLGs (Table 1), with 1–15 RAD loci being associated per peLG. In particular, synteny was detected with all 21 groups of stickleback (gaGroup). In three cases, two common pandora LGs were linked to a stickleback LG, *i.e.*, peLG13 and peLG24 to gaGroupI, peLG2 and peLG23 to gaGroupIV, and last peLG14 and peLG3 to gaGroupVII. In the latter case, peLG3 had only a single hit to gaGroupVII. Overall, conservation of synteny was more apparent in stickleback compared to medaka.

For Nile tilapia (*n* = 22), we also identified one-to-one homology for almost all peLGs with Nile tilapia LGs (onLG), sharing 1–19 homologous loci per LG (Table 1). In particular, 15 out of 24 peLGs were homologous to a single onLG. It is worth noting that both peLG6 and peLG19 showed full homology to onLG7. Further, peLG24 showed no homology to any onLG. Overall, common pandora seemed to share higher homology with Nile tilapia than with stickleback.

**Figure 4:**
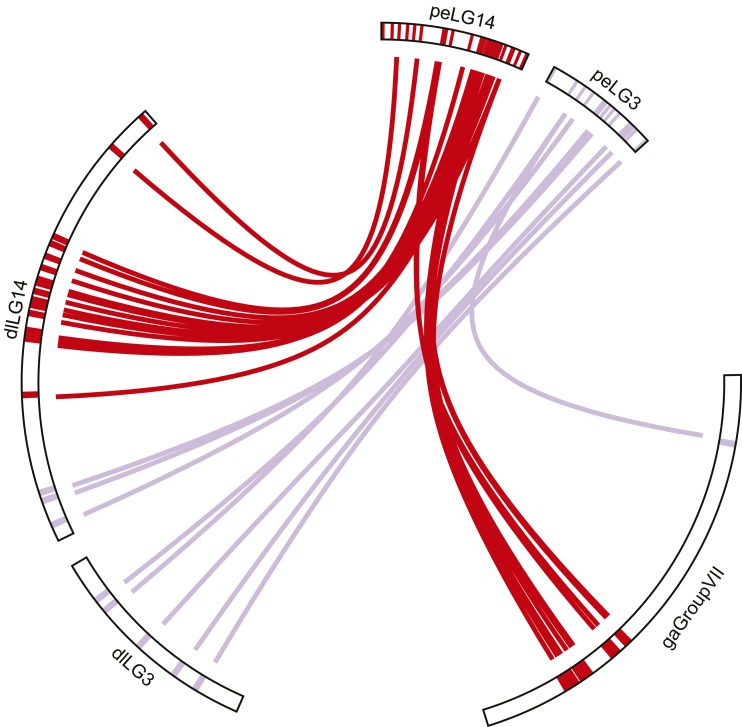
Detailed comparative view of common pandora peLG14 and peLG3 in comparison to stickleback gaGroupVII and European seabass dlLG3 and dlLG14. Links show homology between pairs of loci. Link colors follow the same pattern as in Figure 3.

Finally, in a comparison with the recently published European seabass genome (*n* = 24), we observed the greatest homology with common pandora RAD loci. Between five and 39 RAD loci were identified in each European seabass chromosome (Figure 2). European seabass had 1-to-1 homology with common pandora for all of its 24 chromosomes, with two European seabass chromosomes (dlLG) containing multiple homologous loci from 2 peLGs (Table 1). First, dlLG1B showed 100% agreement to peLG21 (*i.e.*, peLG21 had no homology with any other dlLG) and to parts of peLG16. Second, dlLG14 showed 100% consistency to peLG14 and to parts of peLG3. Note that both peLG14 and peLG3 were also assigned to stickleback gaGroupVII. To understand further the syntenic relationships among peLG14, peLG3, gaGroupVII, and dlLG14 and dlLGB1, we plotted their homology pattern in Figure 4. The pattern observed shows that while peLG14 is clearly homologous to dlLG14 and gaGroupVII, peLG3 has a weaker signal. However, it is linked with both dlLG14 and dlLG3 in European seabass, and only with gaGroupVII in stickleback.

### Phylogenetic reconstruction

**Figure 5:**
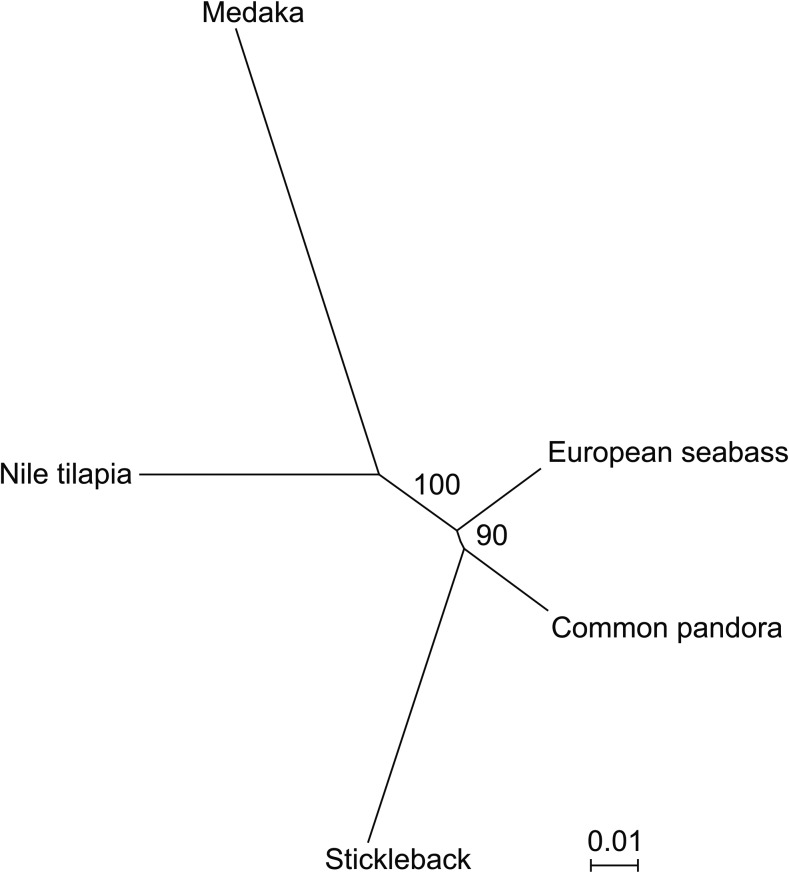
RAD-based maximum likelihood phylogenetic tree of common pandora and the species used in the comparative analysis. The tree was built using HKY+ Γ_4_ model on the concatenated alignment of 50 loci shared among all five species. Bootstrap values are shown as branch labels.

Tree reconstruction took place after aligning the 50 RAD loci that had a hit in all four species used for the comparative analysis. From those loci, 41 were coding while the rest were noncoding. The concatenated alignment consisted of 5015 nucleotide sites, where 3752 were well-aligned and used for the phylogenetic reconstruction employing the model HKY + Γ_4_. The resultant unrooted phylogenetic tree suggested a close relationship of common pandora to stickleback (BS = 90) (Figure 5). European seabass clusters with the common pandora/stickleback clade (BS = 100) and Nile tilapia is closer to medaka.

## Discussion

The employed modified ddRAD methodology allowed the discovery of nearly 1000 polymorphic loci in the unexplored genome of common pandora. Based on 97 progeny of one full-sib family, we built a linkage map of 24 linkage groups, potentially corresponding to the 24 chromosomes of the species, and compared them with those of medaka, stickleback, Nile tilapia, and European seabass, revealing various levels of genome conservation.

### ddRAD sequencing as a linkage mapping tool

The ddRAD methodology applied is an adaptation of the first ddRAD protocol (Peterson *et al.* 2012), slightly modified from Palaiokostas *et al.* (2015). Rather than processing each sample separately or in small groups with final pooling after amplification, the strategy used was to pool all samples after ligation of barcoded adapters, such that only a single gel size-selection step was required. Library production was thus much less labor-intensive than the original ddRAD protocol, and produced a common set of fragment sizes for all samples. Although read depth per individual varied considerably (possibly due to unevenness in initial DNA quality and quantification), the data produced were more than adequate to infer a robust linkage map for the screened pedigree. A major advantage of linkage mapping by genotyping by sequencing *cf*. individual SNP or microsatellite assays is the opportunity it provides to use the sequence data generated to explore similarities to other genomes, as shown in previous studies (*e.g.* Amores *et al.* 2011; Recknagel *et al.* 2013; You *et al.* 2013; Kakioka *et al.* 2013).

### Common pandora linkage map

The constructed linkage map included 24 LGs matching the expected number of chromosomes given that the haploid genome of common pandora—and of all other already karyotyped sparids—is organized in 24 chromosomes (Cataudella *et al.* 1980). This prior knowledge guided our LOD score choice for the linkage computations assisting our data analysis design. The methodology applied for the linkage map construction is based on the average between sexes, taking advantage of the informative markers detected for both parents, but without taking into account the differences in recombination between the two.

Given the importance of integrating the knowledge provided by previous QTL mapping efforts, we indirectly identified the candidate LGs of common pandora that are likely to accommodate QTL found in Gilthead seabream based on the linkage map of Tsigenopoulos *et al.* (2014). As most studies have used stickleback for comparative genomic analyses, we used our comparative genomic analysis with stickleback (see below) as means to bridge the LGs built in each study with the present map. Based on the findings presented in each study regarding the homology of built Gilthead seabream linkage groups (SpLG) with stickleback genome, we can assume the location of several QTL in the linkage map of common pandora (see Table S3). This linkage map is expected to guide future QTL mapping experiments and selected breeding programs.

### Comparing the genome of common pandora with other teleosts

We analyzed the homology of the generated common pandora linkage map with other teleosts based on presumed homologous RAD loci. The linkage map contained numerous polymorphic markers that exhibited significant sequence similarity with other teleost genomes. Notably, our comparative analysis revealed large syntenic regions within the other four species examined (Figure 3 and Figure S2). The built peLGs tended to have one-to-one homology with chromosomes of European seabass, Nile tilapia, medaka, and stickleback. The extensive synteny and chromosome homology of common pandora with these species suggest a relatively conserved genomic structure among the groups, and provide an independent line of evidence confirming the robustness of the employed linkage mapping approach.

Medaka, a species with 24 chromosomes, showed the least similarity to common pandora, indicative of a more distant evolutionary relationship between the two species. However, most common pandora LGs were matched to a single main homologous chromosome in medaka genome, except peLG21, where no homology was observed. Note that peLG21 has the least homology in all comparisons, possibly due to the low number of included loci. Only peLG16 seems homologous to a combination of different medaka chromosomes. The rest exhibit strong homology with one medaka chromosome, apart from two LGs containing only a single homologous locus in medaka. Overall, although the amount of similarity is limited, common pandora LGs have apparent synteny with medaka chromosomes.

Stickleback (*n* = 21) has the least number of chromosomes of the examined species and is known for its rapid divergence from other teleosts in terms of chromosome number and morphology (Urton *et al.* 2011). The observation in this study that six common pandora LGs exhibited homology to three stickleback LGs in a pair-wise manner is consistent with expectations, reflecting the fusions that have taken place in the ancestral stickleback genome. This has been similarly observed in other synteny studies involving sticklebacks, *e.g.*, in the comparison of platyfish linkage map to stickleback (Amores *et al.* 2014), and in the comparative analysis of the European seabass genome with stickleback (Tine *et al.* 2014). Stickleback chromosomes GroupI, GroupIV, and GroupVII are formed by the fusion of ancestral chromosomes that share homology to peLG13-peLG24, peLG2-peLG23, and peLG14-peLG3, respectively. The same three stickleback chromosomes seem to be homologous to the European seabass pairs dlLG13-dlLG24, dlLG2-dlLGX, and dlLG14-dlLG3 in the analysis of Tine *et al.* (2014). The agreement of our results with Tine *et al.* (2014) and the homology of common pandora LGs with single chromosomes in all medaka, Nile tilapia, and European seabass confirm the independence of common pandora LGs. Conservation of synteny between sparids and stickleback had been revealed by previous efforts to conduct comparative mapping in gilthead seabream (Sarropoulou *et al.* 2007; Kuhl *et al.* 2011; Tsigenopoulos *et al.* 2014).

Comparison to Nile tilapia revealed a high degree of conservation of synteny. Nile tilapia is known to have 22 chromosomes—two less than common pandora. Thus, assuming that the ancestral teleost had 24 chromosomes, one would expect to observe two fusion events in Nile tilapia compared to common pandora. Indeed, peLG6 and peLG19 both link to onLG7, a known fused chromosome in tilapia (Guyon *et al.* 2012). Interestingly, the second fusion event could not be detected in our comparative analyses. A denser linkage map is needed to more clearly resolve the syntenic relationship between common pandora and Nile tilapia.

European seabass shared the highest sequence homology with common pandora. The majority of the 24 European seabass linkage groups shared homology with a single common pandora LG. Exceptions were dlLG1B and dlLG14, which showed homology to two peLGs. Particularly for dlLG14, we observe homology with peLG14 and peLG3 (Figure 4). Surprisingly, those two LGs correspond to the fused stickleback gaGroupVII. However, peLG3 shares homology also with European seabass dlLG3. Thus, we can hypothesize that there is a translocation of a chromosomal part of seabass from dlLG3 to dlLG14. Note that dlLG3 is one of the smallest chromosomes of European seabass genome. To independently confirm this observation, we tracked down the homology of European seabass dlLG3 and dlLG14 with stickleback in the deep synteny analysis conducted by Tine *et al.* (2014). In this screening, we confirmed the link between dlLG3-dlLG14 and that of two more European seabass LG pairs observed to be linked in stickleback genome in our analysis (dlLG13-dlLG24, dlLG2-dlLGX) as well. Thus, if we assume that Nile tilapia, medaka, and common pandora maintained the ancestral structure of linkage groups peLG3 and peLG14, we can hypothesize a translocation in European seabass toward the direction of the fusion observed in stickleback.

Overall, from our comparative genomic analysis, we found no common pandora LG that shares homology with more than one chromosome in all the compared species, confirming the linkage mapping conducted.

Finally, the percentage of common pandora RAD loci identified as being homologous to coding regions of the reference species was relatively high (more than one-third on average, Table 2). This possibly reflects the higher chance of finding sequence similarity in the functionally constrained protein coding moiety of the genome compared to noncoding regions, and/or could be due to the ddRAD strategy employed, where restriction enzymes with GC-rich recognition sites were employed in library construction.

### Insights into Sparidae phylogenetic position

The phylogenetic relationship of Sparidae to other teleost families is still controversial (reviewed in Hanel and Tsigenopoulos 2011). The similarity search of RAD loci *vs.* Nile tilapia, stickleback, medaka, and European seabass indicated that common pandora, and probably Sparidae as a whole, was more similar to European seabass than any of the other model species (sharing twice as many RAD loci as the second most similar species—Nile tilapia). This finding does not appear to be unduly biased by differing levels of genome completeness among the four model species, with the CEGMA pipeline analysis confirming similar coverage across all. However, our phylogenetic analysis suggests a closer relationship of Sparids with stickleback rather than European seabass or Nile tilapia with relatively high support (BS = 90). Given this analysis outcome, one would expect a higher sequence similarity of common pandora with stickleback, which was not observed. The fact that common pandora has higher number of similar sequences throughout the genome with European seabass and Nile tilapia compared to stickleback might reflect the longer branch, and thus the higher sequence divergence, observed in the latter (Figure 5). Further, the phylogenetic analysis puts forward that European seabass is closer to stickleback *cf*. Nile tilapia. This is in line with the findings of Tine *et al.* (2014) who resolved the phylogenetic relationship of European seabass with model teleost species through a rigorous phylogenomic analysis of 621 genes. In the phylogenetic tree constructed by Tine *et al.* (2014) stickleback exhibited a profoundly longer branch compared to European seabass. Finally, medaka and tilapia clustered together agreeing with their known phylogeny (Betancur *et al.* 2013). However, the clustering of common pandora with stickleback in the ddRAD markers phylogenetic analysis suggests that the translocation observed in European seabass LG14-LG3 (Figure 4) is independent from the chromosomal fusion that led to stickleback GroupVII, regardless of the involvement of the same linkage groups in common pandora. To answer this question, and unambiguously resolve the position of sparids in the tree of teleosts, a thorough phylogenomic analysis with broad taxon sampling and inclusion of multiple informative outgroups has to be employed.

### Conclusions

Here, we built the first linkage map for common pandora and present the first application of RAD-Sequencing in the Sparidae family. This linkage map should provide the basis for future marker-assisted selection and QTL mapping of important traits on the species, boosting its aquaculture production through genetic selection programs. Moreover, the extensive similarity of common pandora genome compared to European seabass could be an indication that the prediction of Direct Genomic Value (DGV) in common pandora broodstock might not be as ineffective (Taylor 2014), when Genomic Estimated Breeding Values (GEBV) are available in a training population of a more commercially exploited aquacultured species (*e.g.*, European seabass, Gilthead seabream).

On top of the importance of our effort for aquaculture, we use common pandora as a starting point to understand in more depth the genomic evolution of sparids. Comparative genomic analyses revealed an extensive conservation of the genome evolution of common pandora mostly compared to European seabass, Nile tilapia, and to a lesser extent to stickleback and medaka. Interestingly, our phylogenetic analysis suggested that the genomic sequence similarity observed does not reflect phylogenetic proximity as common pandora seems phylogenetically closer to stickleback rather than to European seabass or Nile tilapia.

Finally, the addition of genome-wide information from new nonmodel species from the enormously big tree of teleost fish will shed light upon the evolution of this diverse group of vertebrates. The inclusion of more and more nonmodel species to the genomics arena is the great opportunity in the postgenomic era, leading to a spectacular increase of the available knowledge on teleosts.

## Supplementary Material

Supporting Information
