# Supplementary material for: Exploring a Nonmodel Teleost Genome Through RAD Sequencing—Linkage Mapping in Common Pandora, Pagellus erythrinus and Comparative Genomic Analysis
Source: G3 (Bethesda). 2015 Dec 28;6(3):509–19. doi: 10.1534/g3.115.023432 (PMC4777114; doi:10.1534/g3.115.023432)
Supplement: Supporting Information [file supp_6_3_509__index.html]

Exploring a Nonmodel Teleost Genome Through RAD Sequencing—Linkage Mapping in Common Pandora, Pagellus erythrinus and Comparative Genomic Analysis — Supporting Information 

# Exploring a Nonmodel Teleost Genome Through RAD Sequencing—Linkage Mapping in Common Pandora, *Pagellus erythrinus* and Comparative Genomic Analysis

## Supporting Information for Manousaki *et al.*, 2016

**Files in this Data Supplement:**

- Figure S1 - Mapping LOD score as a function of the number of linkage groups in the resulting linkage maps. (.eps, 2064 KB)
- Figure S2 - Comparative view of common pandora linkage groups with Nile tilapia (left), stickleback (middle) and medaka (right). (.eps, 4603 KB)
- Figure S3 - Venn diagram of the common loci in the comparative analysis of common pandora against European seabass, Nile tilapia, stickleback and medaka for (top) all loci and (bottom) only those that belong to coding regions. (.eps, 1957 KB)
- Table S1 - Sequencing reads assigned to each individual after demultiplexing and length filtering, and stacks summary data. (.xls, 52 KB)
- Table S2 - Stacks loci used for linkage mapping: sequence, mapping and comparative genomic information. (.xls, 477 KB)
- Table S3 - Common pandora linkage groups that correspond to Gilthead seabream linkage groups accommodating QTL based on homology with stickleback. (.xls, 30 KB)
- File S1 - The R script used to build the linkage map. (.R, 1 KB)
